# Cerebral microhaemorrhage count is related to processing speed, but not level of symptom reporting, independently of age, psychological status and premorbid functioning, after first-ever mild traumatic brain injury

**DOI:** 10.1007/s11682-023-00788-0

**Published:** 2023-06-30

**Authors:** Jacqueline F.I. Anderson, Lana Higson, Michelle H. Wu, Marc L. Seal, Joseph Yuan-Mou Yang

**Affiliations:** 1 Melbourne School of Psychological Sciences, The University of Melbourne, Melbourne, VIC 3010 Australia; 2 Psychology Department, The Alfred hospital, Commercial Rd, Melbourne, VIC 3004 Australia; 3 Medical Imaging, The Royal Children’s Hospital, Melbourne, VIC 3052 Australia; 4 Developmental Imaging, Murdoch Children’s Research Institute, Melbourne, VIC 3052 Australia; 5 Department of Paediatrics, The University of Melbourne, Melbourne, VIC 3010 Australia; 6 Neuroscience research, Murdoch Children’s Research Institute, Melbourne, VIC 3052 Australia; 7 Neuroscience Advanced Clinical Imaging Service (NACIS), Department of Neurosurgery, The Royal Children’s Hospital, Melbourne, VIC 3052 Australia

**Keywords:** Mild traumatic brain injury, Cognition, Susceptibility weighted imaging, Recovery, Outcome

## Abstract

Cerebral microhaemorrhage is a commonly identified neuropathological consequence of mild traumatic brain injury (mTBI) and can be identified in vivo using susceptibility weighted imaging (SWI). This study aimed to determine whether SWI-detected microhaemorrhages are more common in individuals after a single, first-ever, mTBI event relative to trauma controls (TC) and to investigate whether a linear relationship exists between microhaemorrhage numbers and cognition or symptom reporting in the post-acute period after injury, independently of age, psychological status and premorbid level of functioning. Microhaemorrhagic lesions were identified by expert clinical examination of SWI for 78 premorbidly healthy adult participants who were admitted to hospital after a traumatic injury and had suffered a first-ever mTBI (n = 47) or no head strike (n = 31). Participants underwent objective cognitive examination of processing speed, attention, memory, and executive function as well as self-reported post-concussion symptomatology. Bootstrapping analyses were used as data were not normally distributed. Analyses revealed that the mTBI group had significantly more microhaemorrhages than the TC group (Cohen’s d = 0.559). These lesions were only evident in 28% of individuals. The mTBI participants demonstrated a significant linear association between number of microhaemorrhages and processing speed, independently of age, psychological status, or premorbid level of functioning. This study shows that a single mTBI causes cerebral microhaemorrhages to occur in a minority of premorbidly healthy individuals. Greater microhaemorrhage count is independently associated with slower processing speed, but not symptom reporting, during the post-acute injury period.

Mild traumatic brain injury (mTBI) that results in hospital treatment is estimated to occur in 300–400/100,000 individuals per year (Voss et al., 2015). Approximately 80% of these individuals are expected to make a full cognitive recovery within 3 months of injury (Carroll, Cassidy et al., 2004). The cognitive domains most commonly affected by mTBI are speed of processing, attention, memory and executive function (Carroll et al., 2014). During the normal recovery period, and for those who do not recover fully in the typical timeframe (Carroll et al., 2014), cognitive impairment significantly contributes to disability and poor psychosocial outcome after injury (Benedictus et al., 2010; Caplain et al., 2017; Ponsford et al., 2003).

Microhaemorrhagic lesions are one of the most commonly identified neuropathological consequences of mTBI (van der Horn et al., 2018) and can be identified in vivo using susceptibility weighted imaging (SWI) (Huang et al., 2015). It has broadly been accepted that groups of individuals with mTBI have more SWI-related microhaemorrhagic lesions than orthopaedic or healthy control participants (Tate et al., 2017; Trifan et al., 2017; van der Horn et al., 2018). The clinical consequences of microhaemorrhagic lesions continue to be debated, however. Some studies have found no significant relationship between the presence of SWI-identified microhaemorrhagic lesions and cognitive function or symptom reporting (Einarsen et al., 2019; Tate et al., 2017; van der Horn et al., 2018). Others have reported a relationship between the presence of microhaemorrhages and adverse neurological and functional outcome (de Haan et al., 2017; Park et al., 2009), cognitive outcome (Huang et al., 2015; Irimia et al., 2022; Studerus-Germann et al., 2018), post-concussion symptoms (Studerus-Germann et al., 2018) and presence of recent onset depression (Wang et al., 2014).

Problematically, all these studies varied substantially with respect to the MRI scanner magnetic field strength and within study scanner consistency, comprehensiveness of outcome evaluation, time since injury, extent of control group matching, inclusion of elderly (> 60 years), presence of psychiatric history and history of previous mTBIs. Further, no previous study controlled for age or premorbid level of cognitive function. All of these variables have been shown to significantly impact microhaemorrhagic lesion presence and/or lesion detection and/or cognition and symptom reporting (Carroll, Cassidy et al., 2004; Haller et al., 2018; Lange et al., 2011; Massey et al., 2015; Ponsford, 2013; Robles et al., 2022; Salthouse, 2009; Stern, 2002; Stulemeijer et al., 2008; Terry et al., 2019; Vernooij et al., 2008). Consequently, the specificity of previous findings with respect to the presence of mTBI-related microhaemorrhagic lesions and the impact of these lesions on cognition and/or symptom reporting remains open to question.

One additional limitation of all prior studies particularly undermines the validity of past conclusions. No previous study that has examined the relationship between microhaemorrhagic lesions and outcome, (Einarsen et al., 2019; Huang et al., 2015; Studerus-Germann et al., 2018; Tate et al., 2017; van der Horn et al., 2018) has investigated or controlled for the potential impact of psychological factors on this relationship. It has been well established that psychological status significantly affects both symptom reporting and cognitive performance after mTBI (Cassidy et al., 2014; Lange et al., 2011; Snell et al., 2015). Consequently, the absence of methodological or statistical controls for the substantial influence of psychological factors on outcome in previous research is highly problematic as it prevents valid inferential conclusions being drawn from these studies.

The present prospective study aimed to investigate post-acute (6–12 weeks after injury) outcome in a premorbidly healthy group of adults, less than 60 years of age, who had suffered a first-ever mTBI, and compare it to a premorbidly healthy group of trauma control adults, who were well-matched for age, sex, premorbid level of functioning, injury cause, involvement in litigation and current psychological status. It was hypothesised that the presence of microhaemorrhagic lesions in the mTBI group would be linearly associated with cognitive function and post-concussion symptomatology after controlling for age, psychological status and premorbid level of function.

## Method

### Participants

Participants comprised individuals, excluding professional athletes and war veterans, who had suffered any traumatic injury (systemic and/or head) between September 2015 and December 2019, and been consecutively admitted to The Alfred hospital or Royal Melbourne Hospital, Melbourne, Australia, in the preceding 6–12 weeks. Detailed description of the recruitment process and the recruitment decision tree have been reported previously (Anderson & Fitzgerald, 2020; Anderson & Jordan, 2020). All admitted trauma patients were approached for recruitment consideration. The mTBI group comprised 47 premorbidly healthy adults (36 male) aged 18–60 years, whose traumatic injury included a head strike and fulfilled criteria for a mTBI event as defined by the World Health Organisation criteria (Carroll, Cassidy, Holm et al., 2004), which can be briefly summarised as (i) 1 or more of confusion or disorientation, loss of consciousness for 30 min or less, post-traumatic amnesia less than 24 h; (ii) Glasgow Coma Scale score of 13–15 after 30 min. Excluded individuals were those with: any previous neurological history, including documented mTBI; any history of heavy alcohol consumption, intravenous or regular Class A drug use; history of any past or current significant psychiatric disorder; current TBI as a result of physical assault/attack; lack of conversational English fluency. The TC participants comprised 31 premorbidly healthy adults (27 male) aged 18–60 years, whose traumatic injury had not included a head strike and who did not report any symptoms of mTBI; this group had the same exclusion criteria as the mTBI group. No ethnic group differences existed. All participants provided informed consent and the project was approved by The Alfred hospital and Royal Melbourne Hospital Human Research Ethics Committees.

### Measures

#### Premorbid cognitive functioning

The Wechsler Test of Adult Reading (WTAR) (Wechsler, 2001) is a word reading task, from which accurate estimates of premorbid intellectual functioning (PreIQ) can be derived in individuals with mTBI (Steward et al., 2017).

#### Processing speed

The Symbol Digit Modality Test – (SDMT) is a measure of processing speed that is sensitive to cognitive impairment after mTBI (McCauley et al., 2014). It requires individuals to provide the correct number that corresponds to a given symbol, according to a reference key at the top of the page. On this version of the SDMT, the final score was number of correct items within 2 min.

#### Attention

The Digit Span subtest from the Wechsler Adult Intelligence Scale – 4th Edition (Wechsler, 2008) is a valid, reliable and widely-used measure of attention that is recommended for use in TBI research (Wilde et al., 2010). Digit Span Total (DSp) is a global measure of attention; raw scores rather than aged-scaled scores were used to enable comparative analyses with other cognitive measures.

#### Memory

The Rey Auditory Verbal Learning Test (RAVLT) (Schmidt, 1996) is a reliable and valid measure of verbal memory (Helmes, 2000). The total number of items learned on the five list learning trials (Total) assessed acquisition; it has demonstrated sensitivity to TBI samples (Schoenberg et al., 2006).

#### Executive function

The difference between Trail Making Tests A and B was used as a measure of mental flexibility (Lezak, 1995). This measure has been shown to be sensitive to executive dysfunction after mTBI (Spreen & Strauss, 1998).

#### Post-concussion symptoms

The Rivermead Post Concussion Symptoms Questionnaire (RPQ) is a widely used measure of post-concussion symptomatology. It assesses physical (10 items), psychological (3 items) and cognitive (3 items) symptoms experienced during the past 24 h, with each item on a 5-point likert scale (0–4) (King et al., 1995). It has been shown to be elevated after mTBI and other conditions (Cassidy et al., 2014; Ettenhofer & Barry, 2012; Laborey et al., 2014).

#### Psychological distress

Two widely used, valid and reliable questionnaires of psychological distress were used: The Inventory of Depressive Symptomatology (IDS) measures severity of overall depression (Rush et al., 1996). The Beck Anxiety Inventory (BAI) measures anxiety symptomatology (Beck & Steer, 1993). To reduce the number of variables and increase the power of calculations, a Psychological Distress Index was calculated by summing the raw score of the IDS and BAI.

#### Assessment of performance validity

The Digit Span (DSp) subtest from the Wechsler Adult Intelligence Scale, 4th Edition (WAIS-IV) (Wechsler, 2008) was used as a measure of effort (Iverson & Tulsky, 2003). Participants were identified as having problematic effort on testing if they failed on the subscales of Age Scaled Score Total (Fail = 5 or less) and Longest Digits Forward (Fail = 4 or less) (Iverson & Tulsky, 2003), which have been shown to have a likelihood ratio that successfully identifies poor effort (Babikian et al., 2006; Schutte & Axelrod, 2013).

### Procedure

Following recruitment on the ward within 1–4 days of injury, participants returned to the hospital for neuropsychological examination and MRI scans, conducted on the same day, 6–10 weeks after injury. Neuropsychological measures were conducted in the following sequence for all participants: SDMT, WTAR, RAVLT, DSp, TMT, RPQ, IDS, BAI.

### SWI data acquisition

Neuroimaging was performed using a 3T MR scanner (PRISMA, Siemens Healthcare) with a 32-channel head coil. The SWI sequence was acquired as part of a longer clinical research protocol in the transverse orientation (TE = 20ms, TR = 29ms, flip angle = 15°, matrix = 202 × 384, FOV 157 × 210; voxel size 0.55 × 0.55 × 1.5mm^3^).

#### Lesion identification

**Fig. 1:**
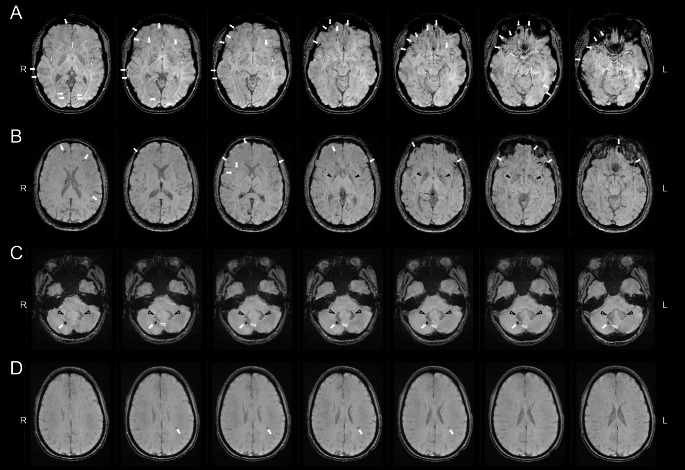
Sample Images of Microhaemorrhages Detected on Susceptibility Weighted Imaging from Selected Study Participants with Multiple Microhaemorrhages *Note*: Image illustrates multi-focal cerebral microhaemorrhages (white arrows) located at the grey-white matter interface and in the deep white matter

Number of SWI-related microhaemorrhagic lesions on each scan was determined concurrently by two raters: a neuroradiologist and a neurosurgical fellow. Each rater had more than 10 years of clinical practice reviewing MRI scans acquired for traumatic brain injury; both raters were blinded to group classification. Any disagreement concerning lesion identification was resolved through consensus. Prior to any consensus discussions occurring, 70% of the sample was randomly selected and correlations were calculated to determine inter-rater reliability of the raters’ decisions. Consistent with previous research (Cheng et al., 2013; Cordonnier et al., 2009; Gregoire et al., 2009) inter-rater reliability in the current study was excellent (r = .99). Cerebral microhaemorrhagic lesions were defined as hypointense foci, less than 10 mm in diameter (Colbert et al., 2010) on SWI data, that were not compatible with vascular flow void (based on sulcal location or linear shape), artefacts from adjacent bone or sinus, or non-haemorrhagic iron/mineral deposition in basal ganglia and other subcortical structures, or as part of a larger intra-parenchymal haemorrhagic lesion (≥ 10 mm) (Greenberg et al., 2009; Nandigam et al., 2009). Sample images from selected study participants are provided in Fig. 1.

If there was doubt as to the aetiology of any foci, the lesions were not considered to be haemorrhagic lesions (Tong et al., 2003). Both raters considered that it was not possible to reliably manually count the absolute number of microhaemorrhages present in cases that had more than 20 microhaemorrhages. Consequently, to conservatively examine group differences, cases that were found to have more than 20 microhaemorrhages were coded as having 20 microhaemorrhages, rather than making estimates of larger microhaemorrhage numbers. A similar approach has been adopted by others when assessing numbers of microhaemorrhagic lesions using SWI images (McKinney et al., 2012).

#### Statistical analysis

Statistical analysis was conducted using the Statistical Program for the Social Sciences (SPSS Version 26.0; SPSS, Inc., Chicago, IL). Data were screened for relevant assumptions for all inferential statistics employed in the analyses. The distribution of microhaemorrhage count was severely skewed. Consequently, consistent with recommended statistical practice for managing non-normally distributed data (Field, 2013), analyses involving the number of microhaemorrhages were undertaken using bootstrapping, with 2,000 samples used in the bootstrapping analyses and bias corrected accelerated confidence intervals identified.

Chi-square tests-for-independence and multivariate analyses of variance (MANOVAs) were conducted to investigate group differences for demographic and clinical variables. MANOVAs were also undertaken to identify any group differences in objective cognition and endorsement of post-concussion symptoms. Bootstrapped t-tests were undertaken to compare groups on the number of microhaemorrhages in each group and bootstrapped partial Pearson correlations were conducted to investigate whether linear relationships existed between cognitive variables and the number of microhaemorrhages. As estimates of premorbid IQ are more accurate indicators of level of premorbid functioning than education (Bright & van der Linde, 2020), premorbid IQ was used as the measure of premorbid level of function.

## Results

The demographic details and injury characteristics for each group are presented in Table 1.

**Table 1:** Demographic and injury variables for mTBI and TC groups

	TC (n = 31) $\stackrel{-}{{X}}$ *(SD)*	mTBI (n = 47) $\stackrel{-}{{X}}$ *(SD)*	p	d*
**Demographics**				
Age (yrs)	37.452 (12.511)	36.192 (14.092)	0.688	0.095
Gender (% F)	12.901	23.400	0.380	-
Education (years)	11.611 (4.860)	12.682 (2.991)	0.822	0.116
PreIQ	105.161 (9.842)	106.085 (9.067)	0.672	0.098
Psych Distress	16.655 (14.482)	15.319 (10.589)	0.644	0.106
Litigation (%)	12.900	14.890	0.111	-
**Injury-related variables (%)**				
Injury Cause			0.730	-
Road trauma	74.110	76.609		
Fall	19.391	19.092		
Sport	6.501	4.299		
GCS [M(sd)]	-	14.513 (0.690)		
LOC (% <5 min)	-	87.201		
PTA (% <60 min)	-	63.800		
Inj to Ax (days)	55.971 (12.670)	62.363 (10.921)	**0.020**	**0.540**

d*: Sensitivity analysis indicated that the MANOVA was sufficiently powered to detect a small effect size (d = 0.3), with power = 0.8; PreIQ: Predicted Full scale IQ; RPQ: Rivermead post-concussion symptom questionnaire; GCS: Glasgow Coma Scale score; LOC: Loss of consciousness; PTA: Post-traumatic amnesia; Inj to Ax: Days between injury and assessment

The groups were well matched on all demographic and injury variables, with the exception of number of days between injury and assessment; the mTBI participants were assessed approximately 9 weeks after injury, whereas the TC participants were assessed, on average, 1 week earlier. The primary analyses of interest were between group comparisons of SWI-detected microhaemorrhagic lesions and within group correlational analyses, neither of which would be affected by this group difference. Consequently, to maximise power, we did not control for this variable. The groups contained equivalent proportions of litigants (13–15%), and one individual failed the assessment of performance validity. This individual was in the TC group, and on inspection was found to have 9 years of education and a predicted premorbid IQ at the cusp of the Borderline and Low Average ranges. It has been suggested that use of Digit Span, as a measure of performance validity, is likely to be less accurate in individuals with Borderline levels of general cognition (Babikian et al., 2006). Consequently, given that the individual’s performance validity status was questionable and they were also in the TC group, making their cognitive performances irrelevant to the primary analyses of interest, they were retained in the sample to maximise power.

Between group comparisons of cognitive performance and endorsement of post-concussion symptoms are shown in Table 2.

**Table 2:** Multivariate analysis of variance results for cognitive and symptom reporting variables

Variable	TC (*n* = 31) $\stackrel{-}{{X}}$ *(SD)*	mTBI (*n* = 47) $\stackrel{-}{{X}}$ *(SD)*	p	*partial η* ^*2*^
SDMT [range]	66.258 (15.576) [34.00–102.00]	66.630 (13.289) [46.00–97.00]	0.911	< 0.001
DSpan Total [range]	27.419 (6.026) [16.00–40.00]	28.553 (4.858) [19.00–39.00]	0.344	0.012
RAVLT T1-5 [range]	51.000 (11.573) [26.00–71.00]	53.064 (9.126) [32.00–71.00]	0.378	0.010
TMT B-A [range]	43.161 (28.516) [6.00–104.00]	35.304 (15.268) [11.00–96.00]	0.121	0.032
RPQ [range]	9.774 (9.972) [0.00–41.00]	9.872 (8.946) [0.00–32.00]	0.989	< 0.001

SDMT: Symbol Digit Modalities Test: number correct; DSpan Total: Digit Span Total; RAVLT T1-5: Rey Auditory Verbal Learning Test, sum of trials 1–5; TMT B-A: Trail Making Test B minus Trail Making Test A; RPQ: Rivermead Post Concussion Symptoms Questionnaire

The groups did not differ with respect to objective cognition in any domain. They endorsed equivalent numbers of symptoms, and effect sizes for all analyses were small (partial *η*^*2*^ < 0.05) or very small (partial *η*^*2*^ < 0.01).

Between group comparison of number of microhaemorrhages identified on SWI revealed that the mTBI group had significantly more microhaemorrhages ($\stackrel{-}{X}$=2.446, SD = 5.853) than the TC group ($\stackrel{-}{X}$=0.129, SD = 0.341) [t(46.47) = 2.708, 95% CI: 0.0879–4.154]; this difference was associated with a medium effect size (Cohen’s d = 0.559). Of note, bootstrapping analyses provide 95% confidence intervals rather than *p* values as indicators of significance, with only those confidence intervals that do not contain 0.00 considered significant (Field, 2013).

**Fig. 2:**
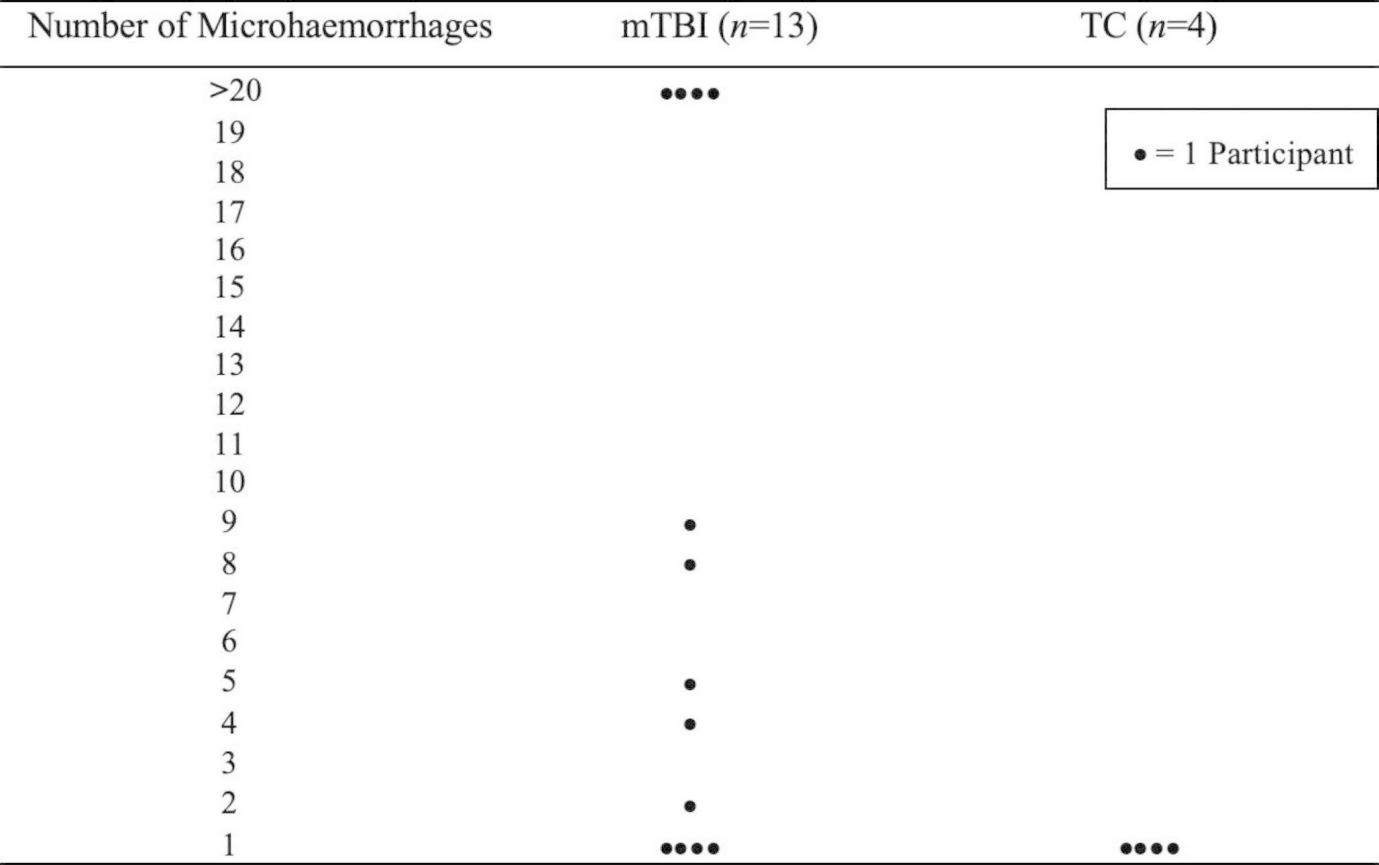
Number of participants per category of microhaemorrhage numbers (> 0) by group

Figure 2 shows the distribution of microhaemorrhagic lesions within the groups for each individual.

For the TC group, 13% of the sample demonstrated microhaemorrhagic lesions, all of whom exhibited only a single lesion. In contrast, for the mTBI group, 28% of the sample demonstrated lesions, with 70% of those with microhaemorrhagic lesions showing more than one lesion and 30% of those with lesions demonstrating more than 20 lesions.

Lobar distribution of microhaemorrhages is presented in Table 3.

**Table 3:** Lobar distribution of microhaemorrhage count by group

		TC *(n = 31)*	mTBI *(n = 47)*
Lobar Location^*^		Microhaemorrhage Count	
Frontal lobe		4	74
	Frontal pole	0	11
	Orbitofrontal gyrus	0	23
	Inferior frontal gyrus (Pars orbitalis)	1	22
	Superior frontal gyrus (prefrontal)	3	6
	Middle frontal gyrus (prefrontal)	0	9
	Precentral gyrus	0	3
Parietal lobe		0	10
	Superior parietal lobule	0	1
	Inferior parietal lobule (SMG)	0	2
	Inferior parietal lobule (AG)	0	1
	Precuneus	0	5
	Posterior cingulate cortex	0	1
Temporal lobe		0	21
	Temporal pole	0	11
	Superior temporal gyrus	0	1
	Middle temporal gyrus	0	4
	Basal temporal (ITG, FG and PHG)	0	5
Insular lobe		0	3
Corpus callosum (genu)		0	2
Cerebellum		0	2

^*^Microhaemorrhages were primarily located at the grey matter-white matter junction, and in the deep white matter. A microhaemorrhage count of 20 was used to represent cases who had great than 20 lesion counts; AG: angular gyrus; FG: fusiform gyrus; ITG: inferior temporal gyrus; PHG: parahippocampal gyrus; SMG: supramarginal gyrus

Lesion distribution primarily involved dorsolateral prefrontal cortex, orbitofrontal cortex, temporal pole and basal temporal lobe, inferior parietal lobule and the precuneus.

**Table 4:** Partial correlations between number of microhaemorrhages and cognitive and symptom reporting variables after controlling for age, premorbid level of functioning and psychological status in the mTBI group

Variable	SDMT r 95% CI	DSpan Total r 95% CI	RAVLT T1-5 r 95% CI	TMT B-A r 95% CI	RPQ r 95% CI
No. microhaem	**− 0.375** **− 0.580 - − 0.154**	0.196 − 0.127 − 0.551	0.057 − 0.187 − 0.405	0.048 − 0.197 − 0.280	− 0.160 − 0.416 − 0.263

SDMT: Symbol Digit Modalities Test: number correct; DSpan Total: Digit Span Total; RAVLT T1-5: Rey Auditory Verbal Learning Test, sum of trials 1–5; TMT B-A: Trail Making Test B minus Trail Making Test A; RPQ: Rivermead Post Concussion Symptoms Questionnaire; 95% CI: 95% confidence intervals are provided in bootstrapping analyses, rather than p-values, with confidence intervals that do not contain 0.00 denoting a significant finding; No. microhaem: Number of microhaemorrhages; Bold: significant finding

After covarying for age, premorbid level of functioning and psychological status, partial correlational analyses were conducted between the number of microhaemorrhages and the cognitive and symptom reporting variables for the mTBI group; these are shown in Table 4.

A significant partial linear correlation between number of microhaemorrhages and speed of processing was evident, with processing speed slowing as the number of microhaemorrhages increased. There were no other significant correlations between the number of microhaemorrhages and other measures of cognition or the symptom reporting variable. The same pattern of significance was evident for the combined mTBI and TC sample, with a significant linear association evident between processing speed and number of microhaemorrhages (r=-.272, CI: − 0.440 - − 0.077). The lack of variance in number of microhaemorrhages in the TC group prevented this analysis from being undertaken with the TC group alone.

## Discussion

This study showed that the mTBI group had greater numbers of SWI-detected microhaemorrhagic lesions than the TC group and that the number of microhaemorrhagic lesions was linearly associated with cognitive function in the head injured group. Specifically, the presence of more microhaemorrhagic lesions was associated with slower processing speed. Location of microhaemorrhagic lesions was primarily in the frontal and temporal lobes, which is consistent with previous findings in individuals with mTBI (Park et al., 2009).

The current finding of greater numbers of SWI-related microhaemorrhagic lesions in those who have suffered a mTBI, relative to a control group, is consistent with recent literature (Tate et al., 2017; Trifan et al., 2017; van der Horn et al., 2018). The present study also replicated a previous finding, which showed that a minority of individuals with mTBI suffer microhaemorrhagic lesions, and that most of these individuals suffer four or more lesions (van der Horn et al., 2018); this contrasts with the single haemorrhagic lesion evident for the small minority of the TC sample that showed any lesion.

The present study demonstrated that there is a direct association between number of microhaemorrhages and speed of processing in the post-acute period after a first-ever mTBI event. Unlike previous research, the current association was found while controlling for psychological status, age and premorbid level of functioning, indicating that the identified association is *independent* of these factors (Massey et al., 2015; Salthouse, 2009; Stern, 2002; Terry et al., 2019). In contrast to expectations, there was no evidence of a similarly independent linear relationship between the number of SWI-detected microhaemorrhages and measures of attention, memory, executive function or post-concussion symptom reporting in either group. Ostensibly, this contrasts earlier findings that have shown relationships between microhaemorrhages and these variables. As previously reported relationships were found without controlling for age, premorbid level of functioning and psychological status, however, past studies cannot address the question of whether independent associations between these variables exist. The current findings indicate that there is no evidence of a linear relationship existing between number of microhaemorrhages and attention, memory, executive function, or post-concussion symptom reporting that is independent of age, psychological status and premorbid level of functioning.

The relationship between number of microhaemorrhages and processing speed, but not other domains of cognition, may be explained by the repeated finding that post-acute changes in processing speed after mTBI have larger effect sizes than changes in other cognitive domains (Frencham et al., 2005). Therefore, any relationship between lesion number and cognition is most likely to be evident in the domain of processing speed. This is supported by the distribution of the lesions in the present study. The location of these lesions corresponds with functional imaging findings that have identified functional and effective connectivity networks that underpin task performance on the SDMT – the measure of processing speed used in this study (Silva et al., 2019). Specifically, it has been shown that networks located in the frontoparietal, temporoparietal and inferior frontal cortices as well as the default-mode network (involving precuneus, posterior cingulate and inferior frontal cortices) underlie SDMT performance. These locations correspond well with the distribution of microhaemorrhages in the current sample.

The lack of relationship between symptom reporting and lesion number is consistent with the post-acute mTBI literature, which has repeatedly demonstrated that psychological status is more predictive of symptom reporting than other measures of injury severity (Cassidy et al., 2014; Silver, 2014). Given the relatively small number of individuals in the current study who had evidence of microhaemorrhages, however, further research examining this question with a larger sample size is warranted.

In sum, although the current study did not demonstrate an average difference in processing speed performance between those with mTBI and those without, there was clear evidence to show that mTBI causes multiple microhaemorrhagic lesions in a minority of individuals. In addition, the minority of those individuals who suffer microhaemorrhages are at significantly increased risk of experiencing slowed processing speed, with increases in the number of lesions associated with greater slowing in processing. It is noteworthy that the significant relationship between processing speed and lesion number for some individuals with mTBI is evident late in the ‘normal’ recovery period (9-weeks post-mTBI), when complete/almost complete recovery is expected for most individuals (Cassidy et al., 2004). The present study also shows that the reduction in processing speed is not due to age, psychological status or premorbid level of functioning. While it is not possible to determine whether the microhaemorrhages directly caused the slowing in processing speed from the current study’s methodology, this possibility clearly warrants future attention. Irrespective of whether a clear causal relationship can be established, however, the current findings suggest that patient care of individuals with mTBI may benefit from clinical management and rehabilitation support that integrate this risk profile in clinical decision-making. This is relevant even when patients are late in the ‘typical’ recovery period.

The primary limitation of the current study was the modest sample size. In particular, the small number of individuals who demonstrated microhaemorrhagic lesions prevented examination of whether there was a predictive relationship between microhaemorrhagic lesion numbers and processing speed performance. Another limitation associated with small sample sizes is the increased risk of making Type II errors. Thus, it is possible that the current study did not identify group differences in cognition due to the modest sample size. Examining the effect sizes of the between group analyses indicates this is relatively unlikely, however, as all effect sizes of non-significant group comparisons were very small. The sample size was considered adequate for bootstrapping analyses (Chernick, 2008), indicating that the partial correlation analyses were unlikely significantly influenced by the modest sample size. A final potential limitation of this study is the possibility that individuals may have suffered pre-traumatic microbleeds, unrelated to the trauma event. Given that the TC and mTBI groups were equivalently pre-morbidly healthy, however, there is no reason to believe that the number of possible pre-traumatic bleeds would have differed between the groups. Consequently, this possibility does not affect the implications that can be drawn from (a) the reported group difference in microhaemorrhagic count and (b) the relationship between processing speed and microhaemorrhagic count.

## Conclusions

In conclusion, this study indicates that a single mTBI seems to cause multiple microhaemorrhagic lesions for a minority of premorbidly healthy adults. Further, for this minority, there is a significantly increased likelihood of experiencing progressively slower processing speed as the number of lesions increases, even at 9 weeks post-injury. While it is not possible to determine if the relationship between number of lesions and processing speed is causal from the current study, the clinical implications of these findings are nevertheless important. Specifically, they raise the question of whether an individual’s mTBI-related microhaemorrhagic lesion load might be an influential factor in the cognitive recovery trajectory after mTBI for some individuals. It is possible that higher lesion load could be associated with slower processing speed and therefore slower cognitive recovery in a minority of individuals with mTBI. Thus, these findings highlight a possible pathological mechanism that might be contributing to variations in cognitive recovery after a single mTBI event in premorbidly healthy adults. Further research will be needed to answer this clinically important question.

## Data Availability

Not applicable.
